# Looking for prognosticators in ovine anaplasmosis: discriminant analysis of clinical and haematological parameters in lambs belonging to differently susceptible breeds experimentally infected with *Anaplasma ovis*

**DOI:** 10.1186/1751-0147-55-71

**Published:** 2013-09-22

**Authors:** Elena Ciani, Ingrid Alloggio, Ferruccio Petazzi, Elisa Pieragostini

**Affiliations:** 1 Dipartimento di Bioscienze, Biotecnologie e Biofarmaceutica, Università degli Studi di Bari, Via Amendola 165/A, 70126 Bari, Italy; 2 Dipartimento dell’Emergenza e dei Trapianti di Organi - Unità di Cliniche Veterinarie e Produzioni Animali, Università degli Studi di Bari, Via Amendola 165/A, 70126 Bari, Italy

**Keywords:** *Anaplasma ovis*, Tolerance to anaplasmosis, Native sheep breeds, Clinical prognosticators

## Abstract

**Background:**

A study was carried out to evaluate the response of different native sheep breeds to experimental infection with *Anaplasma ovis*, the most prevalent sheep tick-borne pathogen in Apulia (Southern Italy). Thirty-four lambs belonging to a Northern European breed (Suffolk) and two Southern Italian breeds (Comisana and Altamurana) were infected. Eleven clinical as well as haematological parameters were monitored at different temporal resolutions on the same subjects before and after the infection, resulting in a data set of 435 observations. The present work, aiming to further the research, presents the results of a multivariate analysis carried out to identify which parameters out of the eleven considered are the most reliable parameters to be considered as markers of the disease phenotype as well as prognosticators of practical clinical importance.

**Results:**

Data were analysed by discriminant analysis. Out of the eleven considered variables (red blood cells, packed cell volume, mean corpuscular volume, mean corpuscular haemoglobin, mean corpuscular haemoglobin content, haemoglobin concentration, white blood cells, neutrophils, leukocytes, platelets, rectal temperature), only seven were included in the step-wise model since significantly increasing the Mahlanobis distance between the two closest groups. Both discriminant functions resulted to be highly significant (P < 0.0001) and the percentage of variation accounted for by the first discriminant function was 63.6% of the variance in the grouping variable.

**Conclusions:**

Taken together, the observed results stress the marked differentiation among the three breeds in terms of physio-pathological phenotypes indicating packed cell volume and red blood cell count as the most informative parameters in the routine clinical practice for *A. ovis* infection in sheep.

## Background

The growing attention to livestock welfare [1] and more environmental-friendly rearing practices is raising the interest in less intensive production systems, that need to be accompanied by a reduction of farm management costs to counterbalance the lower productivity and be economically sustainable and competitive. A first step in this direction may be represented by an effective choice of the genetic material best adapted to the specific environmental conditions ("the right animals in the right place"). Italy is characterized by a complex orography, a wide extension in latitude and a rich history of genetic mixing with animals brought by migrant or occupying populations through the centuries; as a results, a plethora of native breeds, that have evolved in parallel to their nosological context, can be found [2].

As for the latter aspect, a relevant differentiation between Northern and Southern Italy is represented by tick-borne diseases (TBDs), which are enzootic only in the southern part of the peninsula. Thus, breeds traditionally reared below the 41° parallel generally display a good tolerance to TBDs [3]. Due to this feature, breeds from these areas are of particular interest to better understand the phenotype "tolerance to TBDs", which is known to be under complex multi-factorial regulation [4,5] and possibly not unrelated to the erythropoietic system [3] as well as to the haemoglobin genetic systems [6,7].

Based on the above considerations, a study was carried out [8] to evaluate the response of sheep breeds native both from Southern Italy (Comisana and Altamurana) and Northern Europe (Suffolk) through an experimental infection with *Anaplasma ovis*, an obligate intraerythrocytic Rickettsia, the most prevalent sheep tick-borne pathogen (TBP) in Apulia (Southern Italy) [9].

## Materials and methods

The experimental work was conducted in accordance with relevant national legislation on the use of animals and protocols for the study were reviewed and approved by the Animal Welfare and Ethics Committee of the University of Bari. Table 1 summarize the adopted sampling scheme as well as the experimental design.

**Table 1:** Experimental design

**Year**	**Step 1**	**Year**	**Step 2**
2009	Search for carriers	2010	Search for carriers
2009	Splenectomization	2010	Splenectomization
2009	Infection of 8 Suffolk and 8 Comisana	2010	Infection of 18 Altamurana

### Lambs

Lambs less than six months of age were purchased and housed at the Medical Clinics of the Faculty of Veterinary Medicine of the University of Bari. Upon arrival at the Faculty of Veterinary Medicine, the animals were weighed and faecal samples were obtained to establish their worm burdens. Feet were checked for foot rot and possible causes of haematological alteration were excluded by anamnestic investigation, clinical observation and paraclinical exams. The animals were dewormed with a broad spectrum anthelmintic. All of them were then housed in a tick proof isolation unit.

### Pathogen and experimental infection

*A. ovis* was isolated from two local splenectomized sheep, the former was the donor for the 2009 lambs and the latter for the 2010 lambs. Pathogen density was estimated on thin blood film obtained by the buffy coat method and expressed as the percentage of infected red blood cells.

Each lamb in the breed groups was inoculated intraperitoneally with 25 ml of blood taken from the donor sheep at the peak of rickettsiaemia (36% and 60% of red blood cells infected respectively).

### Clinical evaluation

Clinical evaluation was done on a daily basis and rectal temperatures were recorded every morning for 8 weeks post infection. Blood and serum samples were collected twice a week during the observation period. Haematological variables were evaluated using a haematology analyzer. Pathogen density was estimated on thin blood film as above described.

On the whole, eleven clinical as well as haematological parameters (Table 2) were monitored at different temporal resolutions on the same subjects before and after the infection, resulting in a data set of 435 observations. For further details on the experimental design see Pieragostini et al. [8].

**Table 2:** Variables and inclusion/removal parameters^*^

**Variables in the analysis**	**Code**	**Tolerance**	**F to remove**	**Min D squared**	**Between groups**
White blood cells	WBC	0.919	36.900	4.411	1 vs. 2
Mean corpuscular haemoglobin content	MCHC	0.633	25.579	3.720	1 vs. 2
Platelets	PLT	0.597	47.374	2.966	1 vs. 2
Mean corpuscular volume	MCV	0.180	17.311	4.105	1 vs. 2
Rectal temperature	T°C	0.856	34.120	3.577	1 vs. 2
Red blood cells	RBC	0.033	62.830	3.756	1 vs. 2
Packed cell volume	PCV	0.054	83.890	3.813	1 vs. 2
**Variables *not *in the analysis**	**Code**	**Tolerance**	**F to enter**	**Min D squared**	**Between groups**
Haemoglobin concentration	Hb%	0.016	3.603	4.931	1 vs. 2
Mean corpuscular haemoglobin	MCH	0.013	0.992	4.961	1 vs. 2
Neutrophils	N	0.879	1.553	4.923	1 vs. 2
Leukocytes	L	0.893	2.175	4.924	1 vs. 2

Legend: Tolerance, proportion of within-groups variance not accounted for by other variables in the analysis. F to remove, F-value of a variable included in the analysis if it was removed from the model; F to enter, F-value of a variable not included in the analysis if it was included in the model; the SPSS default thresholds for the F-to-enter and F-to-remove were adopted. Min D squared, Mahalanobis distance between the two closest groups. In our study, maximizing the minimum Mahalanobis distance was the variable selection rule while the statistical significance of the F-statistics was used as criterion to enter or remove the selected variables.

^*^Only data from the last step of the inclusion/exclusion process are presented.

## Results and discussion

After inoculation with *Anaplasma ovis*, all the animals developed the disease but symptoms and haematological and clinical parameters varied in terms of severity and duration and none died [8].

Host responses in the three experimentally infected sheep groups were first compared mainly according to typical high fever periods, microscopic observation and haematological values. *A. ovis* began to appear in the blood a week before the fever and the following records showed that erythrocytes infected by *A.ovis* in any case did not exceed 2%.

*A. ovis* infection did not seem to seriously affect Altamurana animals, generally displaying a moderate normochromic normocytic anemia followed by a normochromic macrocytic pattern, suggestive of an active regeneration phase. Conversely, both Suffolk and Comisana animals exhibited a violent response to *A. Ovis* associated to a severe anaemia. However, a slower regeneration was observed in the Suffolk lambs, although they exhibited hyperchromic and macrocytic anaemia, compared to the Comisana lambs that had an hypochromic normocytic anemia. Seven subjects, out of the eight Suffolk animals, needed a conventional treatment with oxytetracycline and dexamethasone every two days for a week to recover, whereas seven subjects out of the eight Comisana animals recovered from clinical anaplasmosis with no treatment other than a single dose of dexamethasone. The highest degree of tolerance was observed in the Altamurana group [8], where all the subjects showed only mild alteration of behaviour and basic life functions in that the animals were only less active, less at ease with their surroundings, less ready to feed and drink.

This work is a step forward from the study described above and its aim was to identify which parameters out of the eleven considered are the most reliable as markers of the disease phenotype as well as prognosticators of practical clinical importance. For this purpose, a discriminant analysis was carried out adopting the step-wise approach implemented in the SPSS v. 16.0.0 [10] statistical package (at each step, the variable that maximizes the Mahalanobis distance between the two closest groups is entered/removed). Discriminant analysis is a multivariate dimension-reduction technique whose main goal is to extract a set of linear combinations of the quantitative variables (discriminant functions) that best reveal the differences among the groups considered [11]. The analysis allows to evaluate the contribution of each variable in discriminating among groups, thus representing an optimal selection tool for the most informative clinical and haematological prognosticators in sheep anaplasmosis. In the step-wise approach adopted here, predictors are entered sequentially. When all variables in the model meet the criterion to stay and none of the other variables meets the criterion to enter, the step-wise selection process stops. The relationships among breed groups were assessed visually by means of a scatter plot with positions of individuals and group means plotted on axes.

In Table 2, the eleven variables, together with their inclusion/removal parameters, are reported. Only seven variables were included in the step-wise model since significantly increasing the Mahlanobis distance between the two closest groups. It must be pointed out that only values observed in the last step of the inclusion/exclusion process are presented in Table 2 which, therefore, does not itemize the whole dynamic inclusion/exclusion process.

Both discriminant functions were highly significant (P < 0.0001) and the percentage of variation accounted for by the first discriminant function was 63.6% of the variance (data not shown).

In Table 3, the standardized discriminant function coefficients, which reflect the contribution of each variable in discriminating among cases, are displayed for the two functions. Considering absolute values, the highest contribution could be ascribed to the two haematological parameters, red blood cell count (RBC) and packed cell volume (PCV), that strongly associated with discriminant function 1 (|3.402| and |3.025|, respectively). The opposite direction of the two variables was not surprising due to the repeated findings of Altamurana as showing a proportionally lower number of erythrocytes, though characterized by larger cell sizes, than North-European sheep breeds [8].

**Table 3:** Standardized discriminant function coefficients

	**Function**	
	**1**	**2**
T°C	-0.045	-0.624
PCV	-3.025	-0.632
RBC	3.402	1.163
MCV	0.514	0.814
MCHC	0.259	0.567
WBC	-0.272	0.540
PLT	-0.183	-0.834

*RT* Rectal Temperature, *PCV* Packed Cell Volume, *RBC* Red Blood Cells, *MCV* Mean Corpuscular Volume, *MCHC* Mean Corpuscular Haemoglobin Content, *WBC* White Blood Cells, *PLT* Platelets.

**Figure 1:**
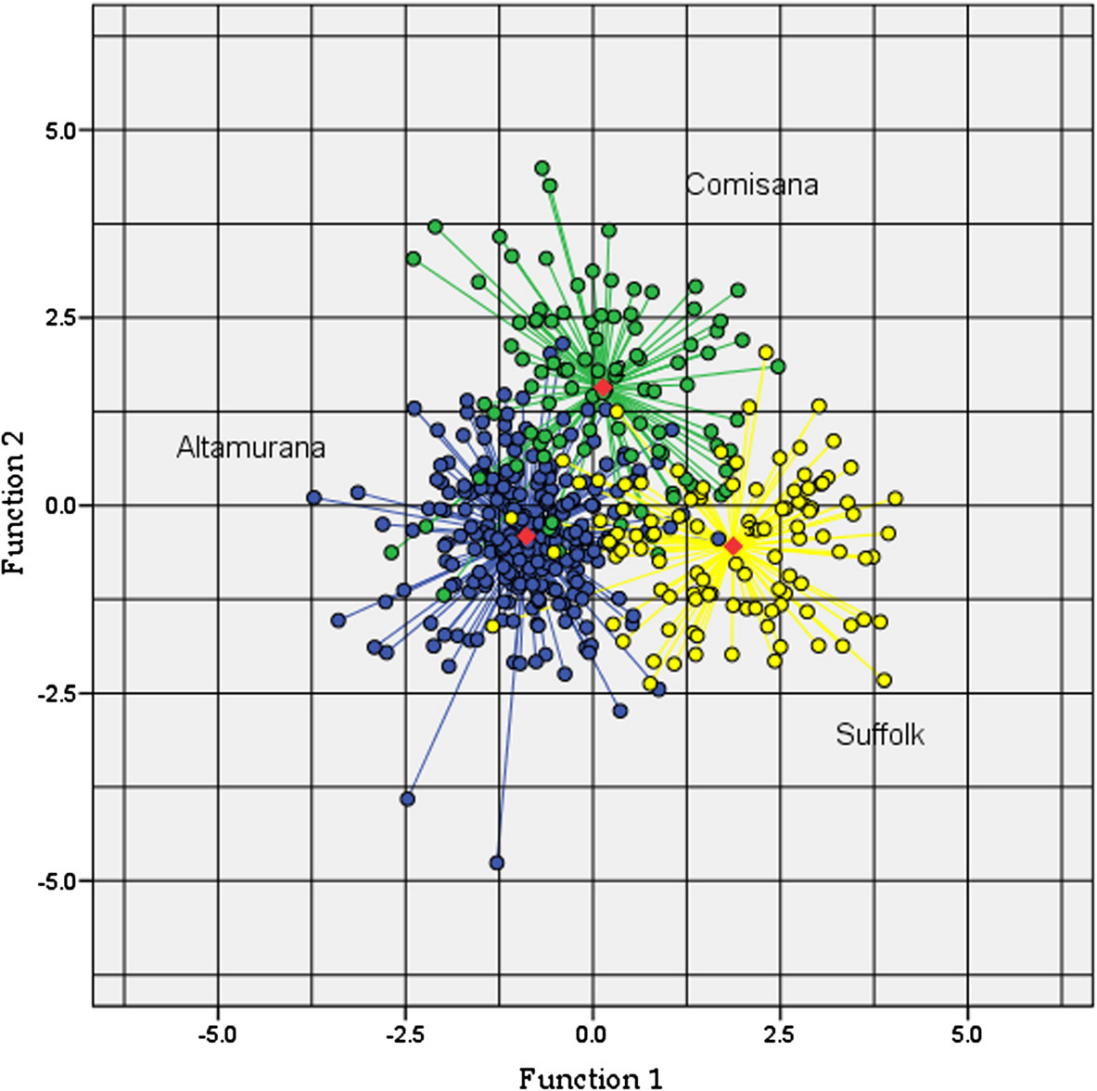
**Scatter plot of the coefficients for the two discriminant functions.** Group centroids are indicated in red.

Once all the seven independent variables were entered, the three groups were shown to be significantly differentiated (P < 0.0001, data not shown), thus confirming the previous results obtained by the univariate analysis which evidenced the different behaviour of the three breeds both in health conditions and in coping with anaplasmosis [8]. The separation of the three breeds was also visually appreciable from the scatter plot of the coefficients for the two discriminant functions (Figure 1).

On the whole, the analysis correctly allocated 83.4% of "cases" to the true breed (Table 4). Altamurana and Suffolk displayed the highest percentages of correct breed assignment (87.6% and 84.6%, respectively), while the Comisana group position in the variables space was intermediate between the former two. Notably, about 28% of Comisana individuals were misassigned, overlapping Altamurana, on one hand, and Suffolk, on the other, with similar percentage values (12.4% and 15.5%, respectively).

**Table 4:** Results of allocation of "cases" according to breed groups^*^

		**Predicted group membership**			
	**Breed**	**1**	**2**	**3**	**Total**
Count	1	205	22	7	234
	2	12	70	15	97
	3	10	6	88	104
%	1	87.6	9.4	3	100
	2	12.4	72.2	15.5	100
	3	9.6	5.8	84.6	100

^*^83.4% of original grouped cases were correctly classified.

Although we cannot rule out the possibility that selection for productive specialization (milk *vs.* meat) may be responsible for the different physiological assessments, environmental adaptation seems likely to have played a major role in shaping the more "controlled" response of the Altamurana lambs, and, to a lesser extent, that of the Comisana lambs, to the experimental infection with a nosological agent enzootic in the area where the breed was developed. As a consequence, the considered breeds may represent a good model to investigate the molecular basis underlying tolerance to *A. ovis*. In particular, the unusual haemoglobin polymorphism recorded in Altamurana [6] as well as the other Apulian native sheep breeds [7,12] and the related functional effects may have an adaptive significance related to the local selective pressure exerted by the pathogen and therefore deserve closer examination.

## Conclusions

Taken together, the observed results confirm the hypothesis of a marked differentiation among the three breeds in terms of physio-pathological phenotypes, with the haematological picture being a relevant feature. Notably, RBC and PCV were the most informative parameters, representing valuable prognosticators in the routine clinical practice for anaplasmosis in sheep.

## Competing interests

The authors declare that they have no competing interests.

## Authors’ contributions

EP and FP have designed the project and helped analyzing and interpreting the results. IA carried out the experimental work and assisted with data collection, data analysis and interpretation. EC carried out the statistical analysis and drafted the manuscript. EP wrote the manuscript which all authors have read and finally approved.
